# Genome-Wide Association Study of Spawning Traits and Candidate Gene Mining in *Portunus trituberculatus*

**DOI:** 10.3390/ijms27198670

**Published:** 2026-09-28

**Authors:** Xiaotong Pan, Shaoting Jia, Dongfang Sun, Yichen Su, Yuchao Huang, Baoquan Gao, Jianjian Lv

**Affiliations:** 1 State Key Laboratory of Mariculture Biobreeding and Sustainable Goods, Yellow Sea Fisheries Research Institute, Chinese Academy of Fishery Sciences, Qingdao 266071, China; 2 National Experimental Teaching Demonstration Center for Aquatic Sciences, Shanghai Ocean University, Shanghai 201306, China; 3 Laboratory for Marine Fisheries Science and Food Production Processes, Laoshan Laboratory, Qingdao 266237, China

**Keywords:** *Portunus trituberculatus*, spawning traits, genome-wide association study, heritability, candidate genes

## Abstract

*Portunus trituberculatus* is a commercially crucial marine aquaculture crab species in China, renowned for its high economic value. Spawning performance is one of the most important target traits in selective breeding programs for this species; however, the genetic architecture underlying this trait remains largely uncharacterized. In the present study, 160 artificially cultivated broodstock individuals were subjected to genomic analysis, with high-throughput liquid chip arrays employed for individual SNP genotyping. By combining these data with phenotypic records of spawning traits, the heritability of the target trait was estimated to be 0.065. A genome-wide association study (GWAS) using a mixed linear model detected nine SNP loci suggestively associated with spawning performance, with an average proportion of phenotypic variance explained (PVE) of 1.96%. One-way ANOVA of these suggestive loci revealed significant differences in phenotypic values among genotype groups, supporting the marker–trait associations; however, because this analysis used the same cohort as the GWAS, it should be considered exploratory rather than independent validation. These suggestive loci were distributed across 8 chromosomes including chromosome 24, 26, 30, 31, 32, 33, 36, and 48. The genetic effect analysis revealed that among the 9 suggestive loci, 8 exhibited additive genetic effects and the remaining 1 locus showed dominant effects. Based on the physical position information of these associated SNPs, 38 positional candidate genes were identified in adjacent genomic regions; 15 of these genes had clear annotation information, including three genes previously documented as reproduction-related: MOXD1, NFAT5, and DDX11. These results delineate the preliminary genetic basis of spawning traits in *P. trituberculatus* and provide important molecular markers and promising candidate genes for breeding improved varieties with high fecundity.

## 1. Introduction

Spawning traits are among the most important breeding characteristics in aquatic animals. Breeding high-fecundity varieties is essential for promoting the large-scale development of aquaculture. As a typical complex quantitative trait, spawning performance is often regulated by multiple genes and molecular pathways [1] and is strongly influenced by environmental factors [2]. Identifying molecular markers and functional genes associated with spawning traits is central to dissecting the genetic and regulatory mechanisms underlying these traits and provides an essential foundation for breeding new varieties with excellent spawning performance.

Genome-wide association study (GWAS) is a method for investigating genotype–phenotype associations at the genome-wide level. It identifies loci associated with target traits across the entire genome, enabling the discovery of key genetic markers and candidate genes regulating traits [3,4]. In recent years, GWAS has been widely applied in fish and crustacean species, mapping candidate genes for important traits such as growth, disease resistance, and stress tolerance [5,6,7,8,9,10].

In the discovery of genes related to spawning traits, GWAS, with its advantages of high throughput and high precision, has become a core technical method for analyzing the genetic basis of target traits and identifying key candidate genes in animals. In livestock research, GWAS has been used to identify important genes related to reproductive traits in pigs [11,12], such as *ESR1*, *ZP3*, and *SOCS2*. In laying hens [13] and Lingkun chickens [14], genes associated with egg number and egg quality, including *GAL*, *TFAP2D*, and *SLIT3*, have been discovered by GWAS. In aquatic species, for example, GWAS was used to localize numerous SNPs associated with fecundity and screened out reproduction-related genes like *Fem-1C* and *RAD1* in the ridgetail white prawn (*Exopalaemon carinicauda*) [15], which are often enriched in pathways such as the Wnt signaling pathway. These studies demonstrate that GWAS is widely used in research on spawning traits in livestock, poultry, and aquatic animals; however, there were no studies reported in *P. trituberculatus*.

The swimming crab (*Portunus trituberculatus*) is an important marine aquaculture crab in coastal areas of China [16]. According to the China Fishery Statistical Yearbook, the annual aquaculture production of *P. trituberculatus* is approximately 100,000 tons, with an output value exceeding 10 billion RMB [17]. Spawning traits of *P. trituberculatus* directly affect the quantity and quality of seedlings and include fecundity, larval output, hatching rate, and metamorphosis rate [18]. Currently, the seedling supply for *P. trituberculatus* relies almost entirely on artificial breeding. However, under artificial cultivation, factors such as inbreeding degree [19] and mating methods affect reproductive performance. Moreover, as the number of artificially bred generations increases, cultured populations generally show declines in spawning traits, such as reduced fecundity and egg quality, constraining the sustainable development of the industry. Therefore, elucidating the molecular genetic basis of spawning traits in *P. trituberculatus* is of great significance.

However, several key gaps remain. First, the genomic heritability of spawning traits in this species is unknown. Second, no genome-wide association study has been performed for spawning performance in *P. trituberculatus*. Third, the candidate genes and molecular pathways underlying spawning capacity remain largely unexplored. Addressing these gaps is essential for molecular marker-assisted selection and genomic selection in this species.

We hypothesized that the spawning index of *P. trituberculatus* is a complex quantitative trait with low-to-moderate genomic heritability and is controlled by multiple small-effect loci. We further hypothesized that candidate genes located near trait-associated SNPs would include genes involved in reproduction, cell division, ovarian function, or neural regulation of reproductive behavior. In this study, we used a chip array to genotype broodstock crabs, combining these data with spawning trait records to estimate the heritability of spawning traits. GWAS was conducted to identify SNPs suggestively associated with spawning traits and to evaluate their genetic effects. Candidate genes were identified based on SNP positions and genomic annotation. Our findings reveal the genetic basis of spawning traits in *P. trituberculatus* and provide an important foundation for the efficient selection of high-yield, high-quality broodstock.

## 2. Results

### 2.1. Phenotypic Analysis of Spawning Index

Phenotypic analysis of the spawning index, based on pre- and post-spawning body weight measurements from 160 *Portunus trituberculatus* individuals, is presented in Table 1. The values ranged from 0.04 to 1.38, with a mean of 0.19 ± 0.15 and a coefficient of variation of 0.79, indicating high variability.

### 2.2. SNP Genotyping and Heritability Estimation

Genotyping of 160 samples yielded raw genotype data for 43,309 SNP loci. After quality control, a total of 36,706 high-quality SNP loci were retained (Table 2). Analysis of SNP density using 1 Mb windows per chromosome showed that the SNPs were relatively evenly distributed across the 53 chromosomes of *P. trituberculatus* (Figure 1), indicating good genome-wide coverage.

To further explore the genetic basis of the spawning index, variance components and heritability analysis for spawning capacity were performed using GCTA (v1.93.2) software (Table 3). The results indicated low heritability for the spawning index, with an estimated heritability of 0.065, suggesting that this trait is under weak genetic control.

### 2.3. Results of Genome-Wide Association Analysis

This study utilized GEMMA (v0.98.4) software to perform GWAS on 36,706 SNPs from 160 *P. trituberculatus* samples using a linear mixed model (MLM). Based on Bonferroni correction, the genome-wide significant threshold was set at 0.05/N (−log_10_(0.05/36,706) = 5.87), but no SNPs reached this threshold. Using the suggestive significant threshold of 1/N (−log_10_(1/36,706) = 4.56), a total of 9 SNPs suggestively associated with spawning index were detected. These were located on chromosomes 24, 26, 30, 31, 32, 33, 36, and 48 (Figure 2). The Q-Q plot showed that the scatter points aligned well with the trend line at the lower end, indicating low false positives and reliable association analysis (Figure 3). One-way ANOVA of these suggestive loci revealed significant differences in phenotypic values among genotype groups (Table 4). However, because these tests were performed in the same cohort used for GWAS, they are exploratory and do not constitute independent validation. Genetic effect analysis revealed that eight of the nine suggestive loci exhibited additive effects, whereas only one showed a dominant effect (Table 4).

### 2.4. Identification of Candidate Genes

ANNOVAR (v20200607) was used to annotate regions 50 kb upstream and downstream of each suggestive SNP. Combined with the *P. trituberculatus* reference genome, 38 positional candidate genes potentially related to spawning capacity were identified, as detailed in Table 5. Chromosome 26 harbored the most genes. Among these, 15 genes had clear annotation information. Functional enrichment analysis of the candidate genes was performed using GO and KEGG databases (Table 6). GO enrichment analysis showed that these genes were mainly enriched in the “nucleic acid binding” term (GO:0003676). KEGG enrichment analysis indicated primary enrichment in the “Chromosome and associated proteins” pathway (K03036), which is related to chromosome structure and function. Given the small number of annotated candidate genes, these enrichment results should be interpreted as exploratory and hypothesis-generating. No multiple-testing correction was applied; therefore, the enrichment results are underpowered and require confirmation in larger gene sets or independent studies. These candidate genes provide important clues for understanding the genetic mechanisms underlying spawning capacity. Further functional validation and mechanistic studies of these genes will provide a theoretical basis for breeding improvement in *P. trituberculatus*.

## 3. Discussion

In livestock and poultry, GWAS has successfully identified reproductive loci and candidate genes, such as *ESR1*, *ZP3*, *SOCS2*, *GAL*, *TFAP2D*, and *SLIT3* [11,12,13,14]. In aquatic animals, GWAS has also been used to dissect fecundity and reproductive traits; for example, studies in *E. carinicauda* identified SNPs and candidate genes enriched in the Wnt signaling pathway [15], and recent aquaculture studies have extended these approaches to genomic selection for complex traits [5,6,7,8,9,10]. However, no GWAS of spawning traits has previously been reported in *P. trituberculatus*. The present study addresses this gap by providing the first genome-wide assessment of spawning index in this species, estimating its genomic heritability, identifying suggestive SNP loci, and annotating positional candidate genes. These findings provide a preliminary framework for molecular marker-assisted selection and genomic selection for spawning traits in *P. trituberculatus*.

Heritability is a core genetic parameter in selective breeding, with its primary value lying in quantifying the relative contribution of genetic factors versus environmental factors to phenotypic variation [20]. Heritability estimates for spawning-related traits vary considerably among aquatic animals. For example, the heritability of average fecundity in *Litopenaeus vannamei* was 0.16 ± 0.06 [21], and the that of average egg volume in tilapia was 0.18 [22]; both are considered low to moderate [23]. In contrast, the heritability estimates for fecundity traits in *E. carinicauda* ranged from 0.45 to 0.52 [15], indicating moderate to high heritability. In this study, the estimated heritability for spawning index was 0.065, which is classified as low. We hypothesize that this may be due to the longer overwintering and spawning cycles of the crabs, which make them more susceptible to environmental effects. However, this explanation remains speculative. Other possible contributors include the limited sample size, the use of a single cohort and environment, measurement error associated with the body-weight-based *SI* proxy, and the possibility that the trait is strongly influenced by environmental effects. Therefore, the low heritability estimate should be interpreted cautiously and validated in larger, multi-environment populations.

Using chip-based genotyping, we obtained 36,706 SNP markers evenly distributed across 53 chromosomes, providing important data support for genome-wide mining of trait-associated markers. The analysis identified 9 suggestive markers, including 7 located in exonic regions, with a mean PVE of 1.96%. One-way ANOVA was subsequently performed to visualize allele-specific phenotypic effects of these suggestive loci within the same population; however, because this analysis was not independent, it should be viewed as exploratory rather than confirmatory. Notably, genetic effect analysis showed that eight of the nine suggestive loci exhibited additive effects. This distribution pattern aligns well with the core rationale of genomic selection (GS). Additive genetic effects are a primary source of genetic variation for quantitative traits and are characterized by their cumulative nature and stable transmission to offspring. In GS breeding, this genetic model provides an essential foundation for accurately predicting Genomic Estimated Breeding Values (GEBV) [24]. Nevertheless, because these loci are only suggestively associated and were not independently validated, their potential utility for GS should be interpreted cautiously and confirmed in larger, independent populations.

Based on the positional information of trait-associated SNPs, 38 candidate genes were identified. Notably, three genes, *MOXD1*, *NFAT5*, and *DDX11*, have been associated with reproduction in humans or other species. In humans, *DDX11* is involved in cell division and DNA repair; its mutation is linked to premature ovarian insufficiency (POI) in females, potentially affecting ovarian function by influencing the stability of oocyte division [25]. Research on *MOXD1* has primarily focused on mice, where it is highly expressed in key brain regions regulating reproductive behavior (SDN-POA, BNSTpr, MePD). These regions directly participate in the regulation of mating, nurturing, and other reproductive behaviors, suggesting that *MOXD1* may indirectly influence reproductive behavior by marking and regulating sexually dimorphic neural circuits [26]. Studies in mice have shown that *NFAT5* promotes the proliferation of ovarian granulosa cells, which are critical supportive cells for follicular development and oocyte maturation; dysfunction of these cells can impair female fertility [27]. Research in vertebrates indicates these genes are directly related to female reproductive development. However, these findings are based mainly on mammals, and functional evidence in crustaceans is lacking. Therefore, the roles of MOXD1, NFAT5, and DDX11 in spawning traits of *P. trituberculatus* should be considered hypothetical and require functional validation. Additionally, many genes without clear annotation information were identified; these might represent genes unique to regulating spawning traits in crustaceans, warranting functional validation through molecular experiments in future studies.

Future studies should include larger and multi-environment populations, independent validation cohorts, higher-density genomic markers, and functional assays such as expression analysis, RNA interference, or gene editing where feasible. Integrating GWAS with transcriptomics, epigenomics, and genomic prediction will help clarify the genetic architecture of spawning traits and accelerate the development of high-fecundity varieties of *P. trituberculatus*.

## 4. Materials and Methods

### 4.1. Experimental Materials and Phenotypic Measurement

The *P. trituberculatus* used in this experiment were from the core breeding population of the Yellow Sea Fisheries Research Institute, Chinese Academy of Fishery Sciences. Broodstock overwintering, seed breeding, and harvest cultivation were conducted at Changyi Haifeng Aquatic Breeding Co., Ltd. (Weifang, China). In November, 160 healthy, vigorous brood crabs without external injuries were selected for indoor overwintering. Each crab was numbered, and corresponding muscle tissue from the second walking leg was sampled for DNA extraction. From March to April the following year, these 160 brood crabs were used for breeding. Owing to the enormous egg clutches of swimming crabs, which typically contain several hundred thousand to several million eggs, direct enumeration of total egg number is both labor-intensive and subject to considerable counting errors. Previous studies have demonstrated a highly significant positive correlation between body weight and fecundity in this species (*p* < 0.01), suggesting that body weight can serve as a reliable proxy for estimating fecundity [28]. In the present study, we adapted the principle of the gonadosomatic index (GSI) [29] to standardize spawning-related traits. Specifically, we calculated the ratio of the difference between pre- and post-spawning body weight to the post-spawning body weight. This weight difference corresponds to the wet mass of the egg clutch, analogous to the gonad mass measured in GSI, while the post-spawning weight is used as the denominator to correct for individual size variation. We term this ratio the spawning index (*SI*), a standardized phenotypic measure of spawning capacity that reflects the reproductive performance of broodstock crabs. We acknowledge that *SI* is a proxy for relative clutch mass rather than absolute fecundity and may be affected by residual water, incomplete egg release, or handling-related variation. The index is calculated as follows:

$$\mathrm{SI}=\frac{\mathrm{Wb}-\mathrm{Wa}}{\mathrm{Wa}}$$

where *SI* is the spawning index, *Wa* is the body weight of post-spawning, and *Wb* is the body weight of pre-spawning.

### 4.2. SNP Genotyping and Quality Control

Genomic DNA was extracted using a magnetic bead-based method [30] with the Magnetic Universal Genomic DNA Kit (DP705, Tiangen Biotech, Beijing, China). The extracted DNA was enzymatically fragmented, end-repaired, A-tailed, and ligated to adapters, then purified and size-selected with magnetic beads. A 200-ng aliquot of this library was hybridized with probes at 50 °C for 16–24 h. Target fragments were pulled down with capture beads, washed stringently to remove nonspecific binders, and finally sequenced after quality control. After sequencing, Fastp (v0.20.1) [31] was used for quality control of the raw sequencing data (Raw Reads) to obtain high-quality Clean Reads. The alignment software BWA (v0.7.15) [32] was used to map the filtered Clean Reads to the reference genome. The resulting alignments were sorted with SAMtools (v1.19) [33], and PCR duplicate reads were marked using Picard (v2.27.5, https://broadinstitute.github.io/picard (accessed on 15 August 2025)). SNP calling was performed with GATK (v4.0) [34], followed by further filtering based on GATK hard-filtering criteria with thresholds of QD < 2.0, FS > 60.0, MQ < 40.0, SOR > 3.0, MQRankSum < −12.5, and ReadPosRankSum < −8.0. VCFTOOLS (v0.1.16) [35] and related software were used to filter and quality-control SNPs. Quality control standards were as follows: retain SNPs with sequencing depth ≥ 6, per-SNP genotype missing rate < 0.1, minor allele frequency (MAF) > 0.05, and conformity to Hardy–Weinberg equilibrium with *p* > 1 × 10^−6^. ANNOVAR [36] software was used to annotate the quality-controlled SNP sites.

### 4.3. Heritability Calculation

Heritability analysis was performed using the GREML (Genomic Restricted Maximum Likelihood) method in GCTA [37] software to calculate the genomic heritability (*h*^2^) of the phenotype. This method is based on a mixed linear model (MLM):

$$y=X\beta +g+\varepsilon ,\;with\;V=A\sigma_{g}^{2}+I\sigma_{e}^{2}$$

where *y* is the n × 1 vector of phenotypic values (n is the sample size). *β* is the vector of fixed-effect coefficients. *g* is a vector of dimension n × 1, following a normal distribution *g*~N(0, $A\sigma_{g}^{2}$). *A* is the genetic relationship matrix (GRM) constructed based on genome-wide SNP markers. *I* is an n × n identity matrix. *e* is the residual effect vector, following a normal distribution *e*~N(0, $I\sigma_{e}^{2}$). After estimating the variance components using the restricted maximum likelihood method, the heritability is calculated as:

$$h^{2}=\frac{\sigma_{g}^{2}}{\sigma_{g}^{2}+\sigma_{e}^{2}}$$

where $h^{2}$ is the genomic heritability, $\sigma_{g}^{2}$ is the genomic genetic variance, $\sigma_{e}^{2}$ is the residual variance, and $\sigma_{g}^{2}+\sigma_{e}^{2}$ is the phenotypic variance.

### 4.4. Genome-Wide Association Analysis

Genome-wide association analysis (GWAS) was performed using GEMMA [38] software, based on a mixed linear model (MLM) to test the association between SNPs and the target trait. The first three principal components (PCs) were included as fixed effects, and the kinship matrix was included as a random effect:

y=Xα+Zβ+Wμ+e

where *y* is the phenotypic trait. *X* is the fixed effect covariate matrix, *α* is the estimated parameter for fixed effects, *Z* is the indicator matrix for SNPs, β is the SNP effect, *W* is the indicator matrix for random effects, μ is the predicted random individual effect, and *e* is the random residual, following *e*~(0, ${\delta e}^{2}$).

Bonferroni correction was applied. The genome-wide significant threshold and suggestive significant threshold were defined as 0.05/N and 1/N, respectively (where N is the number of SNPs used in the association analysis). Manhattan and Q–Q plots were generated from the GWAS results using the R package (version 4.4.2) CMplot (version 4.5.1). The proportion of variance explained (PVE) for each SNP was estimated using the method provided in the literature [39], with the formula:

$$\mathrm{PVE}=\frac{2{\hat{\beta }}^{2}\mathrm{MAF}(1-\mathrm{MAF})}{2{\hat{\beta }}^{2}\mathrm{MAF}(1-\mathrm{MAF})+{(\mathrm{se}(\hat{\beta }))}^{2}2\mathrm{NMAF}(1-\mathrm{MAF})}$$

where β is the SNP effect from GWAS output, se is the standard error of the SNP effect, MAF is the minor allele frequency in the population, and N is the sample size of the population.

As an exploratory post hoc analysis, one-way ANOVA was used to compare trait values among genotypes at each suggestive SNP. Because this analysis was conducted in the same cohort used for the GWAS, it should not be regarded as independent validation.

Based on significant SNPs, additive (a) and dominant (d) effects were estimated using a single-locus linear regression model. For each SNP, alleles were defined as the reference allele (A_1_) and the effect allele (A_2_). The three genotypes (A_1_A_1_, A_1_A_2_, A_2_A_2_) were coded as: Additive coding: A_1_A_1_ = 0, A_1_A_2_ = 1, A_2_A_2_ = 2, reflecting the dosage effect of the A_2_ allele; Dominant coding: A_1_A_1_ = 0, A_1_A_2_ = 1, A_2_A_2_ = 0, reflecting the deviation of the heterozygous phenotype. A linear regression model was constructed with the spawning traits phenotypic value as the dependent variable:

$$y=\mu +ax_{a}+dx_{d}+e$$

where *y* is the individual phenotypic value, *μ* is the population phenotypic mean, *a* is the additive effect value (*a* > 0 indicates A_2_ is the favorable allele), *d* is the dominance effect value (*d* > 0 indicates dominance advantage, *d* < 0 indicates dominance disadvantage), and *e* is the random error, following *e*~N(0, $\sigma^{2}$).

ANNOVAR was used to annotate the SNP loci identified by GWAS. Based on the reference genome sequence and corresponding annotation file, genes within 50 kb upstream and downstream of each significant SNP were screened, and their functional annotations were obtained.

## 5. Conclusions

In this study, genome-wide genotyping revealed that the spawning index of *P. trituberculatus* has low heritability. We also conducted the first GWAS of spawning traits in this species and identified suggestive SNP loci, most of which exhibited additive effects, together with positional candidate genes. These findings provide preliminary insight into the genetic architecture of spawning traits and suggest that genomic selection may be feasible in principle; however, the identified loci are only suggestive and lack independent replication and functional evidence. Therefore, they remain far from practical application. Independent replication in larger populations and functional validation is essential before these markers can be used in breeding programs.

## Figures and Tables

**Figure 1:**
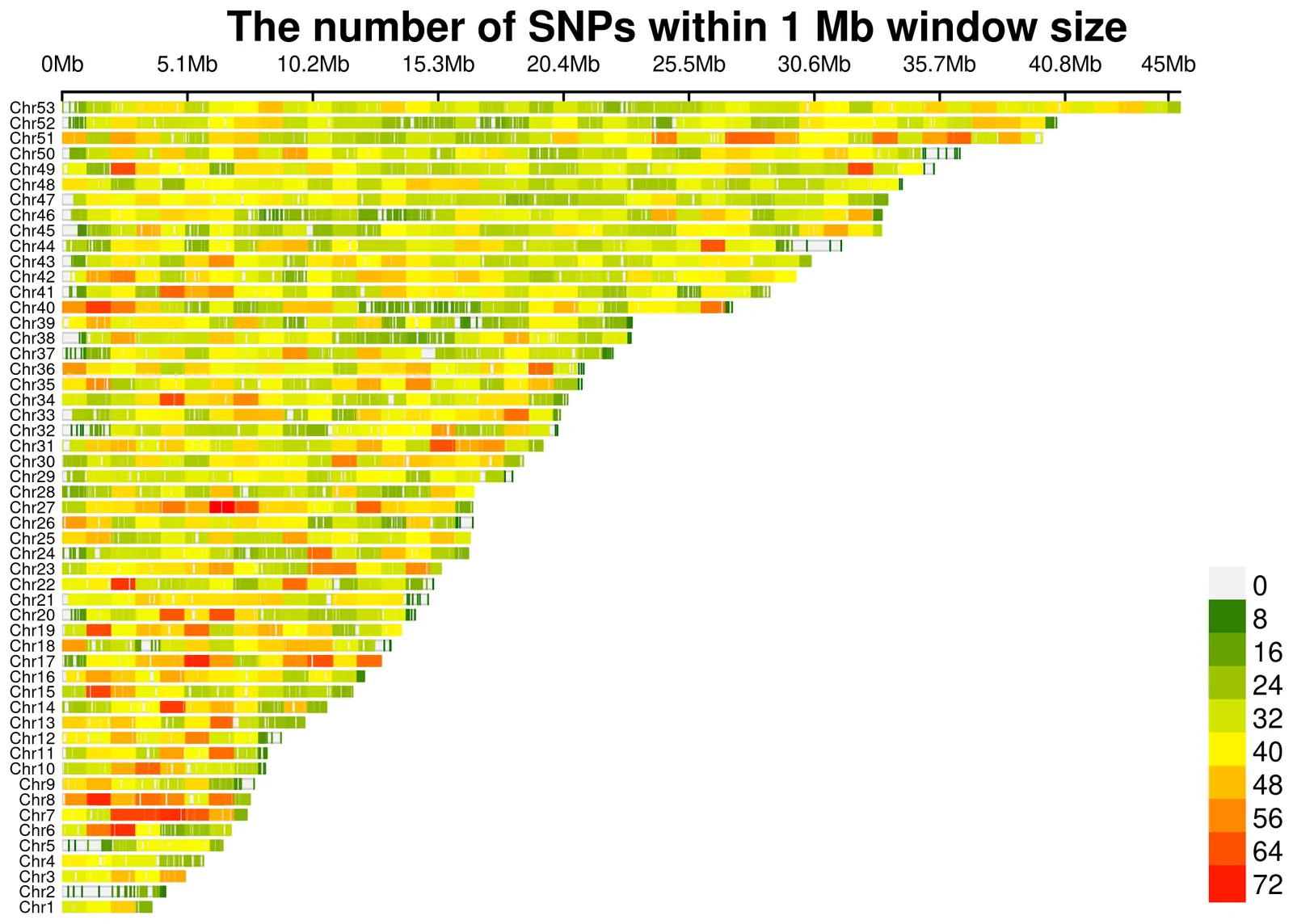
Number of SNPs within a 1 Mb window size on the *P. trituberculatus* genome. The *x*-axis represents the chromosome position (0–45 Mb), the *y*-axis represents the chromosome number (Chr1–Chr53), and the color scale indicates the SNP count within each window.

**Figure 2:**
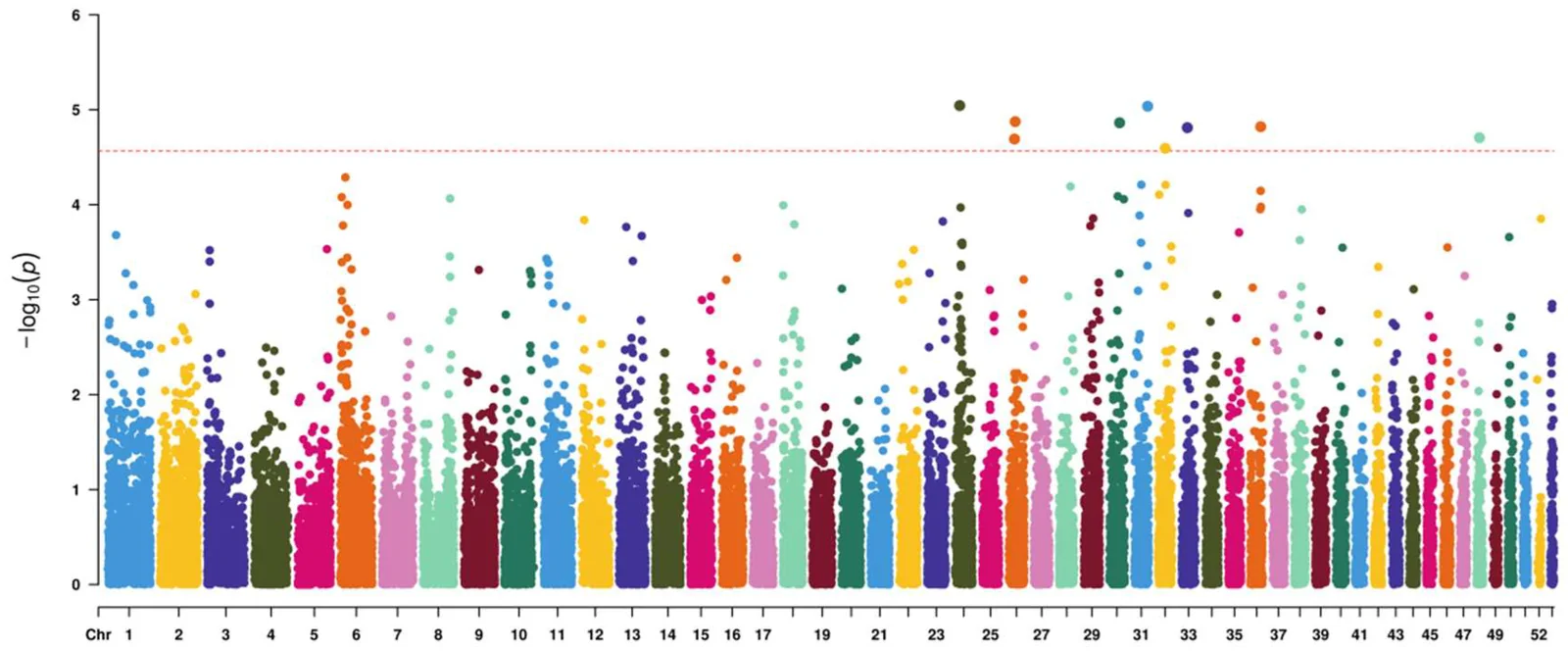
Manhattan plot of the genome-wide association analysis for spawning index of *P. trituberculatus*. The plot illustrates the distribution of −log_10_(*P*) values (*y*-axis) across the 53 chromosomes (*x*-axis). Each dot represents a single SNP. The red dashed horizontal line indicates the suggestive significance threshold (1/N; −log_10_(1/36,706) = 4.56).

**Figure 3:**
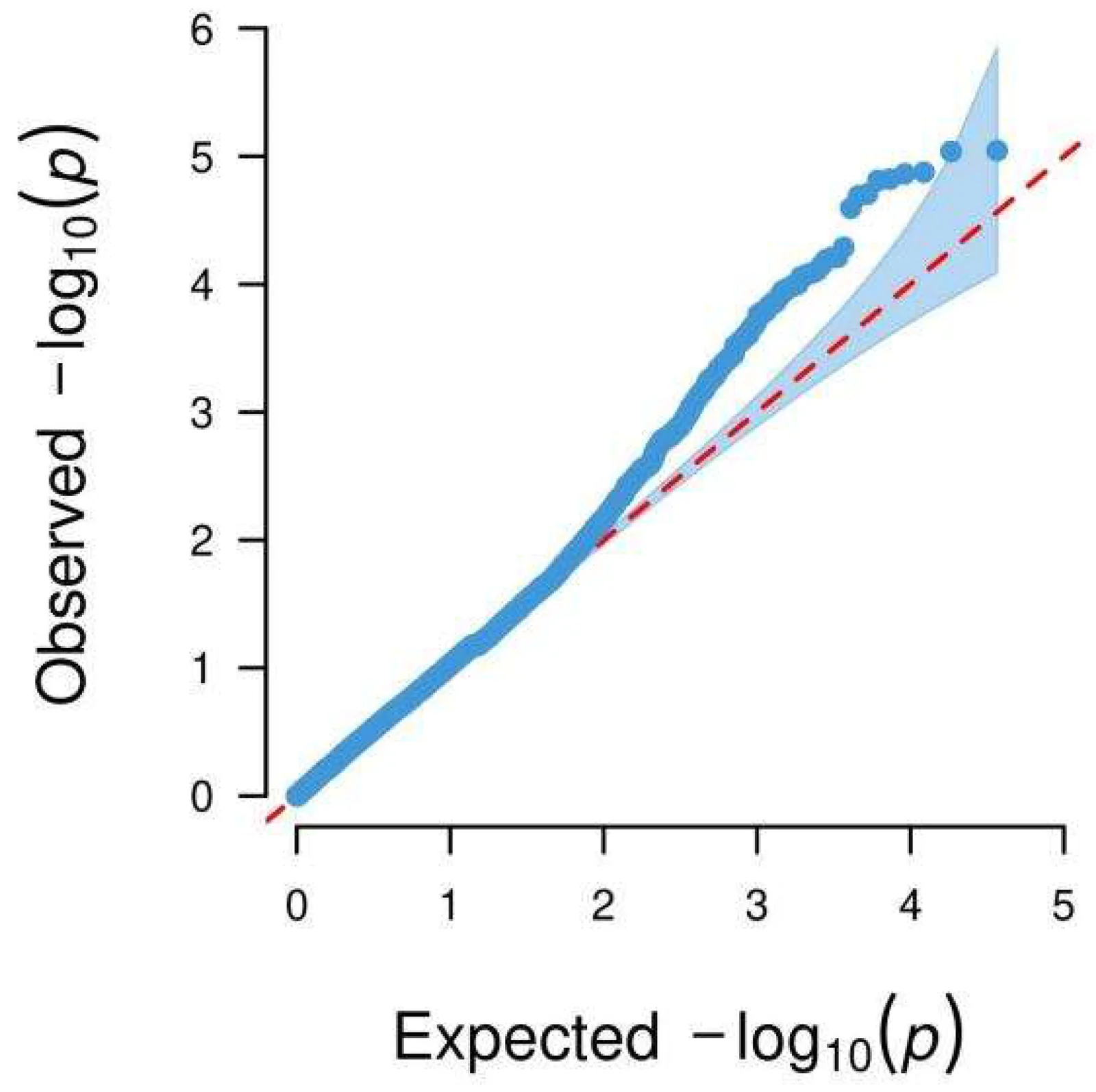
QQ-plot of genome-wide association of total spawning index of *P. trituberculatus*. The *x*-axis shows the expected −log_10_(*P*) under the null distribution, and the *y*-axis shows the observed −log_10_(*P*). The red dashed diagonal line represents the null hypothesis. The blue shaded area indicates the 95% confidence interval.

**Table 1:** Phenotypic descriptive analysis.

Trait	Minimum	Maximum	Mean ± SD	Coefficient of Variation
Spawning index	0.04	1.38	0.19 ± 0.15	0.79

**Table 2:** SNP statistics and annotation results.

Category	Number of SNPs
downstream	1534
exonic	13,886
intergenic	7344
Intronic	8884
splicing	204
upstream	1805
upstream; downstream	1289
UTR3	1177
UTR5	588
UTR5; UTR3	1
Total	36,706

**Table 3:** The heritability of spawning index of *P. trituberculatus*.

Trait	Genetic Variance	Environmental Variance	Phenotypic Variance	Heritability
Spawning index	0.001	0.021	0.023	0.065

**Table 4:** Table of Information on Suggestively Associated SNP Loci.

SNP	Location	PVE	Genetic Effect	Genotype	Number of Individuals	*p*-Value (ANOVA)
Chr24:5047114	intergenic	2.06%	Additive	TT	98	0.003
				TC	45	
				CC	17	
Chr26:6171568	intronic	1.90%	Additive	TT	142	<0.001
				TA	18	
				AA	0	
Chr26:6970868	exonic	1.98%	Additive	TT	143	<0.001
				TG	17	
				GG	0	
Chr30:11184093	exonic	1.98%	Additive	AA	122	<0.001
				AT	35	
				TT	3	
Chr31:14478185	exonic	2.06%	Additive	TT	79	0.002
				TC	63	
				CC	18	
Chr32:7671565	intergenic	1.85%	Additive	CC	102	<0.001
				TC	46	
				TT	12	
Chr33:6064474	exonic	1.95%	Additive	CC	131	<0.001
				TC	24	
				TT	5	
Chr36:10951655	exonic	1.96%	Additive	TT	127	<0.001
				TA	25	
				AA	8	
Chr48:3287571	exonic	1.90%	Dominant	GG	140	<0.001
				GA	18	
				AA	2	

**Table 5:** Candidate Genes Associated with spawning index in *P. trituberculatus*.

Chromosome	Gene	Description	Gene Symbol
Chr24	evm.TU.Chr24.186	-	-
Chr26	evm.TU.Chr26.142	-	-
Chr26	evm.TU.Chr26.143	Oxidoreductase activity, acting on paired donors, with incorporation or reduction of molecular oxygen, reduced ascorbate as one donor, and incorporation of one atom of oxygen	MOXD1
Chr26	evm.TU.Chr26.144	Belongs to the mitochondrial carrier (TC 2.A.29) family	SLC25A12
Chr26	evm.TU.Chr26.145	-	-
Chr26	evm.TU.Chr26.179	ankyrin repeats	ANKLE2
Chr26	evm.TU.Chr26.180	Sequence-specific DNA binding transcription factor activity. It is involved in the biological process described with regulation of the associated gene	NFAT5
Chr26	evm.TU.Chr26.181	-	-
Chr26	evm.TU.Chr26.182	Sulfatase	ARSH
Chr26	evm.TU.Chr26.183	-	-
Chr26	evm.TU.Chr26.186	double-strand/single-strand DNA junction binding	MSH2
Chr30	evm.TU.Chr30.370	-	-
Chr31	evm.TU.Chr31.574	nucleic acid binding	-
Chr31	evm.TU.Chr31.576	N(4)-(Beta-N-acetylglucosaminyl)-L-asparaginase	AGA
Chr31	evm.TU.Chr31.577	ribosomal protein L23	MRPL23
Chr31	evm.TU.Chr31.578	Endoribonuclease activity. It is involved in the biological process described with rRNA transcription	RNASEK
Chr31	evm.TU.Chr31.580	ATP-dependent RNA helicase	DHX33
Chr33	evm.TU.Chr33.226	It is involved in the biological process described with nucleobase-containing compound metabolic process	DDX11
Chr33	evm.TU.Chr33.227	-	-
Chr33	evm.TU.Chr33.228	Meckelin (Transmembrane protein 67)	-
Chr33	evm.TU.Chr33.229	-	-
Chr33	evm.TU.Chr33.230	proteasome regulatory particle assembly	PSMD10
Chr33	evm.TU.Chr33.231	-	-
Chr33	evm.TU.Chr33.232	-	-
Chr33	evm.TU.Chr33.234	Ribose-phosphate pyrophosphokinase 1,2	PRPSAP1
Chr36	evm.TU.Chr36.437	-	-
Chr36	evm.TU.Chr36.438	-	-
Chr36	evm.TU.Chr36.439	-	-
Chr36	evm.TU.Chr36.440	G8	PKHD1L1
Chr36	evm.TU.Chr36.441	IPT/TIG domain	-
Chr36	evm.TU.Chr36.442	-	-
Chr36	evm.TU.Chr36.443	-	-
Chr48	evm.TU.Chr48.184	-	-
Chr48	evm.TU.Chr48.185	-	-
Chr48	evm.TU.Chr48.188	-	-
Chr48	evm.TU.Chr48.189	-	-
Chr48	evm.TU.Chr48.190	-	-
Chr48	evm.TU.Chr48.191	Prenyltransferase activity. It is involved in the biological process described with menaquinone biosynthetic process	UBIAD1

**Table 6:** Integrated Results of KEGG and GO Enrichment Analysis.

Enrichment Type	Pathway/Function Name	Enriched Gene List	*p*-Value
KEGG	Chromosome and associated proteins (03036)	evm.model.Chr33.226, evm.model.Chr33.227, evm.model.Chr36.441, evm.model.Chr33.231, evm.model.Chr33.232, evm.model.Chr48.189, evm.model.Chr36.437, evm.model.Chr36.438, evm.model.Chr26.179, evm.model.Chr33.228	4.8 × 10^−4^
GO	nucleic acid binding (GO:0003676)	evm.model.Chr33.226, evm.model.Chr30.370, evm.model.Chr31.580, evm.model.Chr26.180, evm.model.Chr26.186, evm.model.Chr31.574	2.41 × 10^−2^

## Data Availability

The raw data generated in this study are not publicly available due to institutional restrictions, but are available from the corresponding author upon reasonable request.
